# Specific Genomic Regions Are Differentially Affected by Copy Number Alterations across Distinct Cancer Types, in Aggregated Cytogenetic Data

**DOI:** 10.1371/journal.pone.0043689

**Published:** 2012-08-24

**Authors:** Nitin Kumar, Haoyang Cai, Christian von Mering, Michael Baudis

**Affiliations:** 1 Institute of Molecular Life Sciences, University of Zurich, Zurich, Switzerland; 2 Swiss Institute of Bioinformatics, Quartier Sorge, Lausanne, Switzerland; Duke-National University of Singapore Graduate Medical School, Singapore

## Abstract

**Background:**

Regional genomic copy number alterations (CNA) are observed in the vast majority of cancers. Besides specifically targeting well-known, canonical oncogenes, CNAs may also play more subtle roles in terms of modulating genetic potential and broad gene expression patterns of developing tumors. Any significant differences in the overall CNA patterns between different cancer types may thus point towards specific biological mechanisms acting in those cancers. In addition, differences among CNA profiles may prove valuable for cancer classifications beyond existing annotation systems.

**Principal Findings:**

We have analyzed molecular-cytogenetic data from 25579 tumors samples, which were classified into 160 cancer types according to the International Classification of Disease (ICD) coding system. When correcting for differences in the overall CNA frequencies between cancer types, related cancers were often found to cluster together according to similarities in their CNA profiles. Based on a randomization approach, distance measures from the cluster dendrograms were used to identify those specific genomic regions that contributed significantly to this signal. This approach identified 43 non-neutral genomic regions whose propensity for the occurrence of copy number alterations varied with the type of cancer at hand. Only a subset of these identified loci overlapped with previously implied, highly recurrent (hot-spot) cytogenetic imbalance regions.

**Conclusions:**

Thus, for many genomic regions, a simple null-hypothesis of independence between cancer type and relative copy number alteration frequency can be rejected. Since a subset of these regions display relatively low overall CNA frequencies, they may point towards second-tier genomic targets that are adaptively relevant but not necessarily essential for cancer development.

## Introduction

Genetic changes such as point mutations, regional copy number alterations/aberrations (CNA) and structural changes (e.g. gene fusion events) are all hallmarks of cancer. CNAs arise as somatic changes in the tumor cell genome through a variety of mechanisms and can be observed in virtually all types of cancer, to a varying extent. So far, the most widely used methods for the detection of CNAs have been chromosomal and array-based Comparative Genomic Hybridization (CGH) techniques [1]–[4]. Localized, recurring CNAs (hot-spots) have been shown to target canonical oncogenes (e.g. duplications/amplifications of the MYC, MYCN, REL loci) or tumor suppressor genes (e.g. deletions of the CDKN2A/B, TP53, ATM loci). Some regional CNAs such as gains on 8q and losses on 3p are present across multiple cancer types, whereas other imbalances may be largely restricted to a limited number of cancer entities [5].

Datasets integrated across multiple cancer types have previously been analyzed, to report regional “hot-spots” of frequent CNAs [5], [6]. In a given set of individual tumor samples, the number and distribution of CNAs varies considerably [5] and this genetic heterogeneity has been used to detect and report co-occurring CNAs [7].

In principle, specific patterns and similarities in the individual and/or disease specific CNA profiles might point to distinct oncogenomic mechanisms acting in different cancer types and specimens, given a sufficiently large number of data points. Indeed, clustering of CNA patterns has been used to identify oncogenomic similarities [5], [8]–[11]. The adaptation of clustering techniques to the analysis of CNA patterns has been subject of previous studies [12]–[14]. With a few exceptions [5], [14], however, sample-based clustering has been the main focus of such studies so far. In contrast, we here explore the clustering of cancer types, not of individual cancer samples.

**Figure 1:**
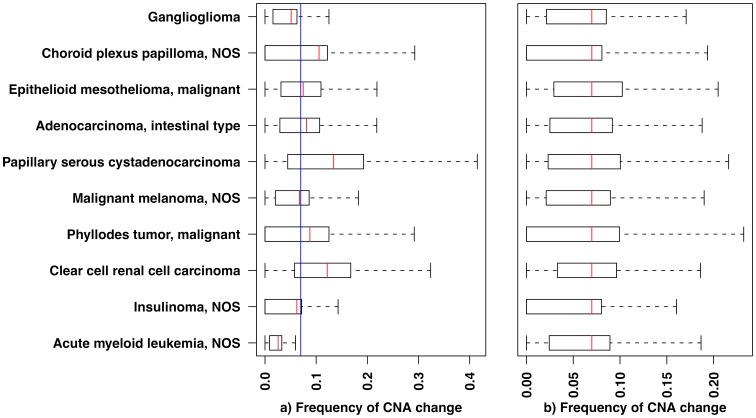
The overall frequency of genomic copy number alterations (CNA) differs among cancer types. Boxplots show the CNA frequency distributions among tumor samples in 10 randomly selected cancer types. The boxplot delineations mark the percentiles 5%, 25%, 75% and 95%. The red lines indicate the mean frequency for each cancer type, whereas the blue line represents the overall mean frequency across all 160 cancer types analyzed here. Frequency values are defined as the ratio of number of samples showing a CNA for a genomic region (i.e., cytogenetic bands) over total number of samples in that cancer type. a) Before normalization b) After normalization. In b) the nominal frequency distribution for each cancer type is re-scaled so that its mean matches the overall mean across all cancer types. (NOS – “not otherwise specified”: high-order classifications, not further assigned to more detailed levels).

Both descriptive and clustering-based analyses of CNA across multiple cancer types suffer from a bias towards the more frequently occurring events. Due to the heterogeneity of the overall CNA signal, with greatly varying average frequencies of CNAs per cancer type (Figure 1a), clustering results may be distorted depending on the disease entities analyzed. This variation in overall CNA occurrence frequencies across cancer types may simply be owed to differences in the average time points of clinical detection or in different progression characteristics, and should be corrected for prior to clustering analyses. To the best of our knowledge, so far no implementation has been reported for a comprehensive, very large-scale clustering analysis of frequency-normalized cancer CNA profiles.

Here, we focus on the identification of genomic regions that contribute meaningfully to the clustering of cancer types. From hereon we will refer to those as “non-neutral” regions. As the starting point of our analysis, we use hierarchical clustering to arrange cancer types on the basis of their CNA frequency profiles. We then employ a permutation approach to estimate the relative contribution of individual genomic regions to the quality of the clustering and to the derived relationship tree. The clustering quality is inferred from an intrinsic measure (summed branch lengths: tree height statistics), and genomic regions that reject the null hypothesis are termed non-neutral. Identified regions are compared to canonical CNA hot-spots (i.e. those that occur most frequently across the entire dataset).

Our current analysis is based on data from a total of 25579 samples, which are classified into 160 different cancer entities (table S1) according to the International Classification of Disease in Oncology (ICD-O 3). Our approach is unique in that it a) focuses less on the clustering as such but more on the individual genomic regions that best support the clustering, b) uses an intrinsic quality measure coupled to a permutation strategy for validation, c) performs CNA frequency normalization prior to analysis, and d) is based on a very large data set, processed in a standardized setup. We aim for the identification of potential cancer-specific driver/modulator regions, which may not have been detected in earlier, largely hot-spot-focused approaches. All of the underlying cancer data is available through our Progenetix repository (www.progenetix.org; [15]).

## Results

The average overall frequency of CNAs across the entire genome varies among different cancer types (Figure 1a). Since the relative weight of CNAs at individual genomic regions in a given cancer type depends on the observed overall genome-wide frequency, we aggregated all patient samples by cancer type and normalized the frequencies of CNAs for each cancer type to the overall mean observed across the entire data set (Figure 1b, Figure S1). The normalized CNA frequency profiles were then clustered using hierarchical clustering.

**Figure 2:**
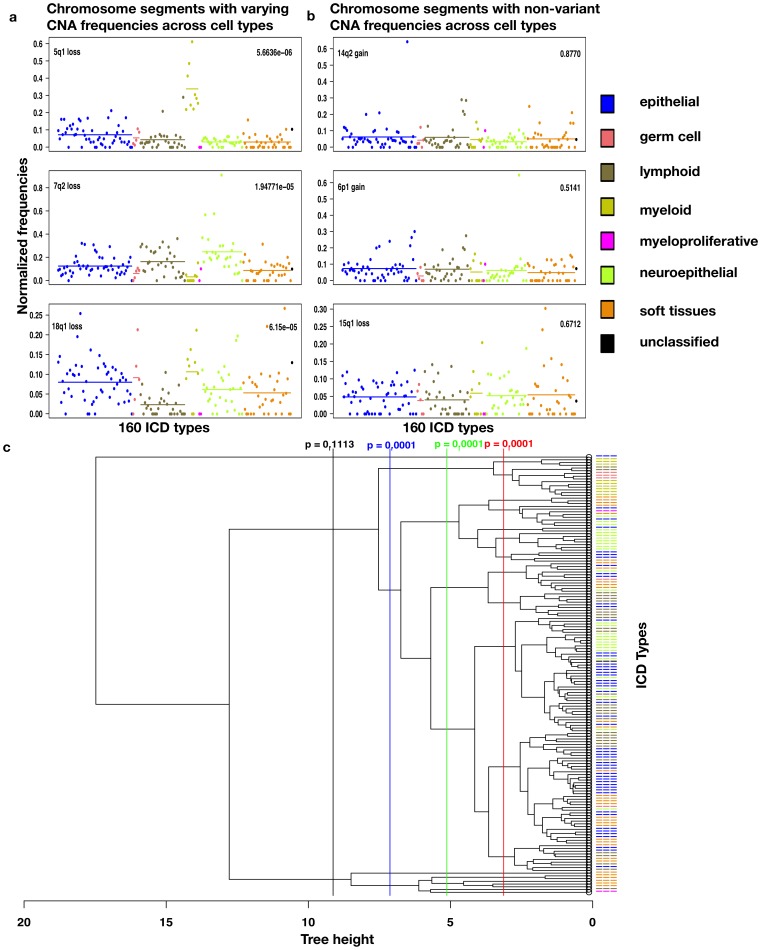
The tissue type of a cancer has a strong influence on its CNA likelihood pattern. a) examples of individual chromosome segments, showing their observed CNA frequencies stratified by cell type. Each dot summarizes all samples classified under one particular ICD type, color-coded by root cell type. In the left panel, three chromosome segments are shown that exhibit strong differences between cell types; on the right, three negative examples without such a signal. All p-values were corrected for multiple testing according to Benjamini-Hochberg. b) the dendrogram (tree) has been obtained using hierarchical Ward clustering on the global frequency-normalized CNA profiles across all 160 genomic regions. Cancer types are again color-coded according to the cell type of origin, with the same legend as in a). Partitioning the tree by cutting at different heights produces multiple clusters; validation of those clusters based on the cancer origin (metric: Random Index) shows that the clustering works significantly better than expected at random.

To evaluate the quality and the biological signal in the clustering, we labeled each cancer type with its “root” cell type (i.e., an undifferentiated cell type from which the tumor likely originated). We expected cancers of the same root cell type to cluster together; this was used as an external proxy for the expected biological relationships between cancer entities. The Random Index [16] was used to compute this external cluster quality measure. Tumors of the same cell type indeed often clustered together, usually in 2–3 small groups (Figure 2). The consistency of this grouping was significantly higher than expected at random, pointing towards biologically meaningful differences in CNA profiles between tumors of distinct origins. Cutting the tree at several heights always led to an observed quality of clustering that was better than the expected random value (Figure 2), except for the cut at the highest level, which resulted in only three clusters. This strongly argues against a completely neutral occurrence pattern of CNAs in the genome, and supports a correlation between biologically meaningful groups of cancer entities and their CNA profiles.

Randomizations of the entire frequency matrix lead to a complete loss of the signal present in the clustering tree (Figure S2), and also strongly reduced the summed branch lengths tree-height statistic.

### Non-neutral CNAs

**Figure 3:**
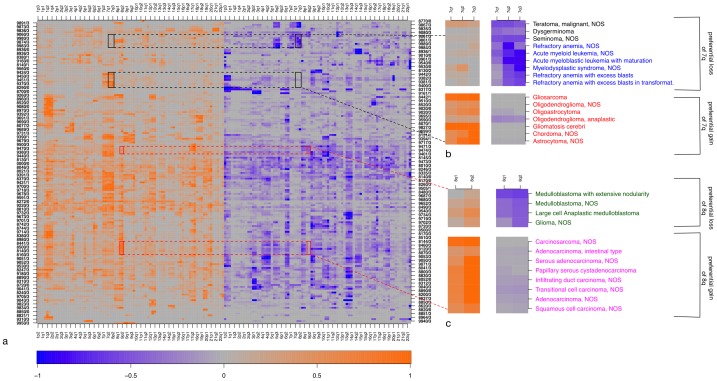
Examples for non-neutral CNA regions. a) Heatmap of CNA profiles on genomic regions (same clustering as in Figure 2). Genomic locations are represented with orange color when considering duplications/gains, and in blue when considering deletions/losses. Color intensity shows relative CNA frequencies; the most-affected region in each row is arbitrarily set the to brightest color (1.0) for display purposes. b) Small regions (black rectangles on the heatmap) are zoomed in to show how non-neutral CNAs can differentiate between cancer types. The example shows that 7q is preferentially gained in brain tumors (red labels) whereas it is preferentially lost in germ cell (black labels), myeloid and myeloproliferative cancer types (blue labels). c) Small regions (red rectangles on the heatmap) are zoomed in to show how 8q is preferentially lost in medullublastomas (green labels) and is preferentially gained in epithelial tumors (pink labels). Some chromosomes consist entirely of non-neutral regions (such as chromosomes 18 and 7). Note that the spatial resolution of the CNA data on the chromosome is limited (roughly corresponding to cytogenetic band resolution).

**Figure 4:**
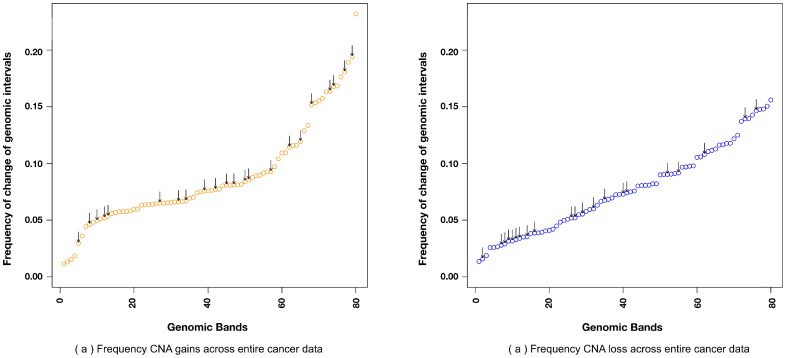
Not only CNA “hotspots” are informative in cancer classification. Genomic regions (bands) are sorted according to their overall frequency of CNAs observed. Those regions that are informative with respect to cancer type clustering are marked with arrows. a) Considering duplications (gains) b) Considering deletions (losses).

**Figure 5:**
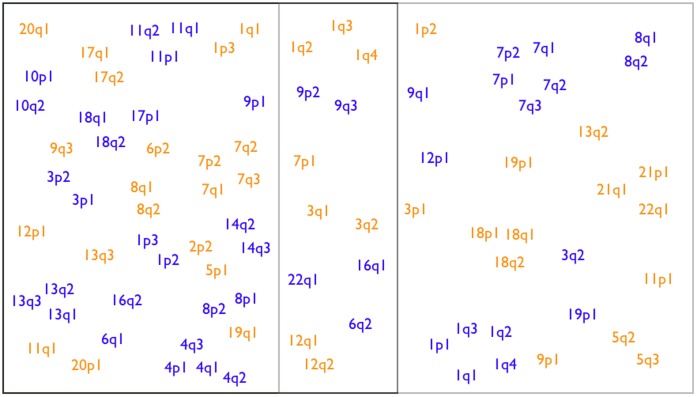
Comparison of non-neutral vs. hot-spot CNA. Genomic regions affected by CNAs, either more frequently than average (black rectangle), or non-neutrally with respect to cancer-type classifications (grey rectangle). The intersection defines regions that are affected both frequently and non-neutrally. Changes are color-coded (gains in orange and losses in blue).

The normalized and clustered frequency matrix encompassing 160 large-scale genomic regions and 160 cancer types is shown in Figure 3. To determine how much each individual genomic region contributes to the overall signal, we individually randomized its profile across cancer types, while keeping the rest of the data unchanged. We then examined the concomitant reduction in the tree length statistics (TLS) of the clustering dendrogram, upon 100000 independent randomizations, to determine the statistical significance of that region's contribution. The resulting cancer-diverging CNA regions are important as they cannot be fully neutral and have the potential to define relationships among cancer types. Indeed, 43 out of the 160 genomic regions (table S1) were observed to have a non-neutral contribution (Bonferroni-corrected p-value 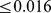) in the aggregated cancer CNA data. Note that gain and loss events were treated independently, and no preferential bias towards gains or losses was observed among the detected non-neutral regions (22 gains and 21 losses). The CNA occurrence frequencies of the non-neutral genomic regions spread thorough the entire frequency spectrum (Figure 4). Only 13 (8 gains and 5 losses) of the non-neutral regions were found altered overall more often than average (Figure 5, intersection of black and grey rectangle), indicating that subset of frequently altered hotspot regions carry a detectable signal to distinguish cancer types (the number of frequently altered regions stands at 59; Bonferroni-corrected p-value 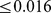, table S1). This observation emphasizes our key point that not only the frequent CNA regions should be used to cluster and annotate cancer types.

22 genomic intervals across 12 chromosomes were found to be informative when specifically considering duplications/gains only (Table 1 and Figure 5). All three genomic segments of chromosome 18 (18p1, 18p2, 18q2) exhibited a signal. For other chromosomes such as chromosome 1 (1q2,1q3,1q4,1p2), chromosome 3 (3q1, 3q2, 3p1), chromosome 12 (12q1,12q2) and chromosome 21 (21p1, 21q1) more than 50% of genomic regions were informative as gains, suggesting simultaneous involvement of multiple loci from these chromosomes. Changes on chromosome 1(1p2), chromosome 3 (3p1, 3q1), chromosome 5 (5q2, 5q3), chromosome 9 (9p1), chromosome 11 (11p1), chromosome 12 (12q1, 12q2), chromosome 18 (18p1, 18q1, 18q2) and chromosome 21 (21p1, 21q1) were selectively informative only as gains. In terms of deletions/losses, 10 chromosomes encompassing 21 genomic regions were found to be non-neutral. Like for chromosome 18 gains, the complete chromosome 7 (7p1, 7p2, 7q1, 7q2, 7q3) was found to be informative when lost (Table 1). Informative regions on chromosome 1 (1p1,1q1, 1q2, 1q3, 1q4) and chromosome 9 (9q1, 9q3, 9p2) covered more than 50% of genomic segments present on these chromosomes. Selective losses were observed on chromosome 1 (1p1, 1q1), chromosome 6 (6q2), 7 (7q1, 7q2, 7q3, 7p2), 8 (8q1, 8q2), 9 (9p2, 9q1, 9q3), 12 (12p1), 16 (16q1). CNAs involving chromosome 1 (1q2, 1q3, 1q4), chromosome 3 (3q2), chromosome 7 (7p1), chromosome 19 (19p1) and chromosome 22 (22q1) were informative both as gain and loss events. This represents a small proportion (16%) of non-neutral CNA. Involvement of a region both as gain and loss may point towards multiple adaptively relevant loci, and/or towards a generally unstable nature of these regions.

**Table 1:** Number of non-neutral regions per chromosome.

Chromosome No.	No. genomic locations	Non-neutral gains	Non-neutral losses
1	7	4	5
2	5	–	–
3	4	3	1
4	4	–	–
5	4	2	–
6	4	–	1
7	5	1	5
8	4	–	2
9	5	1	3
10	3	–	–
11	3	1	–
12	3	2	1
13	4	1	–
14	4	–	–
15	3	–	–
16	3	–	1
17	3	–	–
18	3	3	–
19	2	1	–
20	2	–	–
21	3	2	–
22	2	1	–

Some chromosomes consist entirely of non-neutral regions (such as choromosomes 18 and 7). Note that the spatial resolution of the CNA data on the chromosome is limited (it roughly corresponds to the cytogenetic banding patterns).

### Cancer Diverging Nature of Non-neutral CNA

To provide few examples of cancer classifying behavior of non-neutral changes, we selected a few of the enriched changes and analyzed them for their specific occurrence in different cancers. An example include cancer entities showing predominant losses versus gains on 7q. Preferential losses involving 7q were observed in germ cell, myeloid and myeloproliferative tumors (Figure 3) whereas neuroepithelial brain tumors (among other entities) preferentially displayed gains on 7q. Losses involving 7q are common in myeloid and myeloproliferative tumors [17]–[20] and are associated with advanced age and resistance to therapies [21], [22]. However, here we show that 7q losses are quite specific to myeloid tumors and promote their selective divergence from other cancer types. 7q losses in germ cell tumors had not been explored in detail [23], [24]. With the accumulation of 7q losses virtually restricted to myeloid/myeloproliferative neoplasias and germ cell tumors and in contrast to chromosome 7(q) gains observed in e.g. neuroepithelial brain tumors, it is tempting to propose involvement of at least one common oncogenetic mechanism acting in these clinically unrelated malignancies.

Chromosome 8q gains can be observed in the majority of cancer entities [5], [6]. However, in our analysis 8q losses were enriched as non-neutral events. Preferential losses involving 8q were present in some brain tumors (e.g. medulloblastoma, Figure 3), separating them from other epithelial tumors. Differences in preferential losses involving 8q separated neuroepithelial tumors in two categories with both having gains on 7q but only one (mainly meduloblastomas) having preferential losses on 8q (Figure S3). Losses involving chromosome 8q across medulloblastomas have been reported by a few [25] studies before. Our analysis shows that 8q losses are selected for in some medulloblastomas and therefore could be important for cancer development/progression. Preferential losses of 8q were also observed in germ cell tumors separating them from other epithelial neoplasias (Figure S4).

As another example of restricted CNA types we also looked for cancers showing gains involving chromosome 18. Follicular lymphomas exhibited specific gains on chromosome 18 where as epithelial tumors prefered to loose chromosome 18 (Figure S4). Chromosome 18 gains are very common in follicular lymphomas and are supposed to provide an alternative mechanism for BCL2 activation [26], [27]. However, here we show that this CNA event statistically separates them from other cancer types.

## Discussion

Our current study represents the largest analysis performed to date on cancer CNA data, with the aim of detecting oncogenomic features that may be specifically associated or enriched in certain subsets of cancer entities. In contrast to gene-centric approaches, our analysis assesses the complete information space of genomic copy number imbalances from whole genome profiling experiments.

Overall, the frequency of CNAs across genomic intervals varied between between 0.01% to 23% (Figure 4). Clustering of cancer types on the basis of their frequency profiles helped to identify a class of underlying molecular signals that is orthogonal to histological classifications or clinical categories (the latter are predominantly driven by the affected organ/tissue). Cancer types vary from each other in their CNA abundance, CNA size spectrum and degree of genomic instability. With respect to genomic coverage, large CNAs are generally frequent in cancer [6] and should not be excluded from statistical analyses of cancer genome patterns. While comparing CNA profiles of cancer types, their complexity and variation in frequencies have to be considered. When correcting for these parameters, regional CNAs defining the divergence of the overall profiles can be delineated.

We performed an analysis of a global cancer CNA dataset, identifying 43 genomic regions on 15 chromosomes as significant for CNA profile divergence in cancer types. Obviously, these changes do not cover the entire spectrum of CNA events in cancer, but define a subset of genomic regions that may have a possibly adaptive link to the distinct biology of various cancer types. These regions overlap rather poorly with hot-spot regions observed in many cancers. This suggests that hot-spot regions, though frequently associated with canonical oncogenes, may not always be very useful in aiding data-driven evaluation of cancer (sub-) types.

Disease specific studies have the potential to detect a representative spectrum of oncogenomic aberrations in the given entities. It can be expected that the cancer type specific regions highlighted with our approach had been discussed in the context of the respective publications. However, with our current study, we aim to provide a new, generalized approach at identifying genomic elements relevant in the genesis of individual cancer entities. Although here showcasing a “global” approach without entity pre-selection, our methodology may prove valuable when targeting relevant genomic separators in limited, biologically related entity sets.

Since the current analysis is based primarily on molecular-cytogenetic data from chromosomal CGH experiments with a spatial resolution of several megabases, only inferred information about the causal genes present in the non-neutral regions could be obtained. With upcoming high-resolution genomic array and/or sequencing data, similar analyses will more specifically define the non-neutral CNAs and can be valuable starting points for an integration of the results with functional pathway frameworks. We have recently announced the creation and public availability of a reference resource for oncogenomic array data (www.arraymap.org [28]), which will serve as starting point for such approaches both from our side as well as from interested members of the research community. Also, although we have focused our current analysis solely on a CNA dataset, our methodology should prove particularly valuable when combined with other sets of related diagnostics (for example point mutation data), whereby the assignment of possible driver genes in the non-neutral regions might become feasible.

## Materials and Methods

### Data

Our study is based on well annotated cancer CNA data from the Progenetix project [5], including a total of 25579 samples analyzed by chromosomal (cCGH; 18708) and array CGH (aCGH; 6871) experiments. The clinical samples had been classified into 160 distinct cancer entities according to the International Classification of Disease codes (ICD). At the time of writing, the Progenetix collection represents the largest resource for annotated, whole genome CNA profiling data in cancer.

For our analysis, regional CNA information across all cancer types was reduced to 80 genomic intervals covering the entire genome with the exception of the sex chromosomes. Gain and loss events were considered separately for the analysis, resulting in a matrix of dimensions 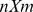, where 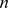 is the number of samples and 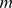 is the number of genomic intervals (*i.e.* 160).

### Cancer Clustering

The frequency of CNA changes across all genomic intervals was computed for each ICD type, and the entire frequency matrix was then normalized (Figure S1). The frequency matrix was ordered using hierarchical Ward clustering. The aggregated separation distance between cancer entities obtained using hierarchical clustering can be analyzed by parsing the clustering tree (dendrogram). The tree represents the relatedness among groups present in the same clade (similar to phylogenetic trees). Randomized data disrupts the tree completely (Figure S2), and the overall tree height statistic is reduced 3-fold, reflecting the complete loss of ordering information present in the original tree.

### Method to Compare Tree Height

We used the tree height as an intrinsic measure to compare cancer associations obtained using clustering and to gauge the information present in the tree; this was used to define non-neutral CNAs. This has advantages over traditional clustering evaluation techniques, as it a) does not require external gold standard information, and b) does not require cutting the tree at an arbitrary distance. The overall tree height is defined as the sum of all direct parent-child relation path lengths in the tree. Tree distances (branch lengths) generally reflect the CNA profile discrepancies between two cancers (or groups of cancers). For any node 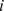, the tree height between this node and its immediate parent 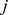 can be measured as 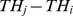. The overall tree height of a tree with 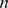 nodes is than obtained as 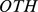  =  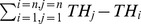 (figure S3).

#### Tree length statistics (TLS)

To identify genomic regions that are non-neutrally affected by CNA we have developed the following permutation strategy:

1. Normalized frequencies of CNA across all genomic intervals are computed across all cancer types.
2. The cancer classification tree is obtained using hierarchical Ward clustering.
3. The observed over all tree height (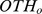) is calculated as mentioned above (Figure S5).
4. A counter 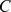 is set to zero for every genomic interval in consideration.
5. For any genomic interval 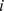, its status values are shuffled among all samples keeping its over all frequency the same (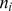).
6. The frequency of CNA at genomic interval 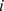 is re-calculated after randomization across all cancer types. The shuffling in the previous step changes the frequency of interval 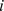 across all cancer types keeping the normalized frequency distribution of all other genomic intervals.
7. The frequencies for interval 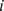 in the normalized frequency matrix from step one are replaced with permuted frequencies for this interval and the permuted overall tree heigh (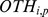) is computed.
8. If 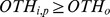, C is incremented as C = C+1.
9. p-value for genomic location 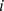, at the end of N (100'000) permutations is computed as 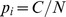.
10. p-values across all bands are corrected for false discovery rate using Bonferroni correction.

### Frequency Based Enrichment (FBE)

Frequently observed CNA regions (“hot-spots”) are genomic changes that occur more often than expected under a fully random null model. Such hot-spot CNAs can be identified using the binomial probability function [29]. Let's suppose genomic interval 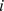 shows a CNA across 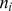 samples out of 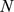 samples. The background CNA frequency (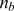) can be represented as the mean frequency change across all intervals. The p value that the frequency of CNA 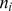, is more than any frequency 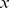 (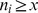) is obtained using the binomial probability function.
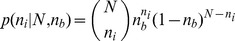


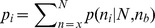



Genomic intervals showing a large deviation from the mean will be assigned low p-values. All p-values are corrected for false discovery rate using Bonferroni correction.

## Supporting Information

Figure S1
**Method for CNA frequency normalization across cancer types.** All the frequencies among cancer types were normalized to the mean frequency of CAN changes across across the 160 cancer types. This normalization was achieved by multiplying the cancer-type-specific frequencies with an index 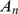, whose value was calculated as shown.(PNG)

Figure S2 **Dendrogram of a permuted frequency matrix.** For this clustering, the frequencies among cancer types were permuted and then normalized. Hierarchial Ward clustering was then performed and the dendrogram tree shown was obtained. The tree height is severely affected by the permutation. In this randomized clustering, similar cancer types no longer clustered together.
(PDF)

Figure S3 **Small regions from heatmap in main** **Figure 3** **are shown here.** These regions represent gains and losses on 7q and 8q. 8q changes differentiate between two categories of brain tumors, with a subset showing preferential losses on 8q (green labels) and other rarely showing involvement of 8q locus (red label). Thus depending on 8q involvement neuroepithelial tumors can be divided in to two different categories. Both of them show 7q gains.
(PDF)

Figure S4 **Examples for non-neutral CNA regions.** a) Heatmap of CNA profiles on genomic regions (same as in Figure 3). b) Small regions (red rectangles on the heatmap) are zoomed in to show how 8q is preferentially lost in in germ cell (black labels) tumors and is preferentially gained in epithelial cancer types (pink labels). c) Small regions (black rectangles on the heatmap) are zoomed in to show how 18q is preferentially gained in medullublastomas (brown labels) and is preferentially lost in epithelial tumors (pink labels). The examples here show that how two different non-neutral changes differential epithelial tumors from germ cell tumors and follicular lymphomas.
(PDF)

Figure S5
**Calculation of over all tree height.** Schematic representation of the summed branch-length tree height statistic. Overall tree height is computed by summing up the distance between all parents and child nodes. Note that the branch lengths of terminal branches (“leafs”) are not considered. Overall tree height  =  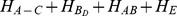.(PDF)

Table S1 **Table with information about cancer types used in the analysis, non-neutral and hot-spot p values.** The table giving details about all cancer types used in this analysis with the corresponding numbers of samples in them and the root cell type of each cancer. The table also has information about the non-neutral and hot-spot p-values obtained for all genomic bands in analysis.
(ODS)
