# Supplementary figures and images for: Specific Genomic Regions Are Differentially Affected by Copy Number Alterations across Distinct Cancer Types, in Aggregated Cytogenetic Data
Source: PLoS One. 2012 Aug 24;7(8):e43689. doi: 10.1371/journal.pone.0043689 (PMC3427184; doi:10.1371/journal.pone.0043689)

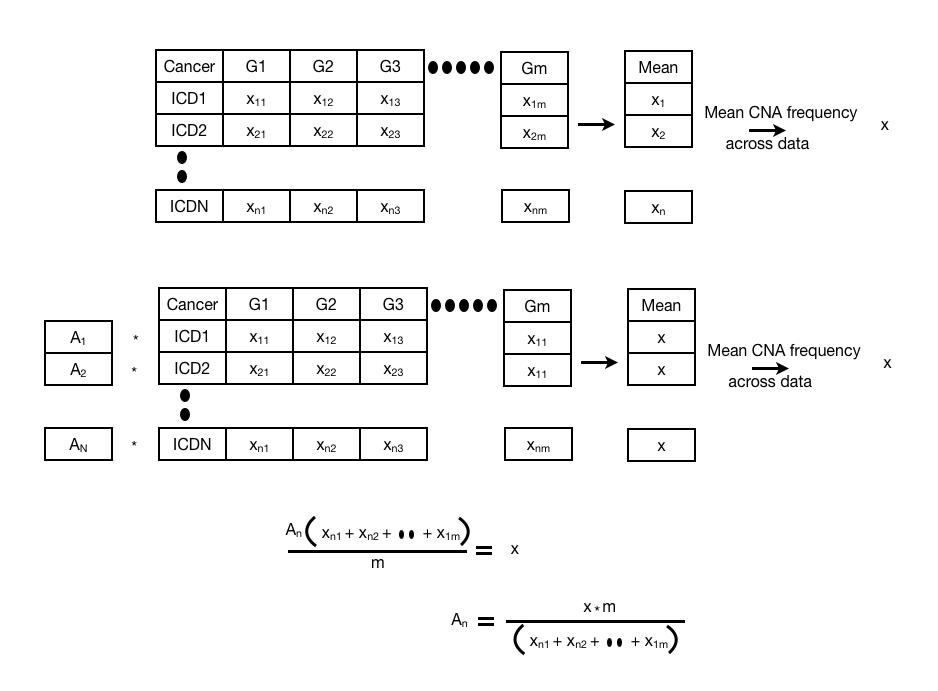

Supplement: Figure S1 — Method for CNA frequency normalization across cancer types. All the frequencies among cancer types were normalized to the mean frequency of CAN changes across across the 160 cancer types. This normalization was achieved by multiplying the cancer-type-specific frequencies with an index , whose value was calculated as shown. (PNG) [file pone.0043689.s001.png]

$H_E$

$H_{AB}$

$H_{A-C}$

$H_{B-D}$

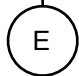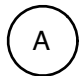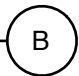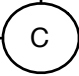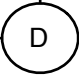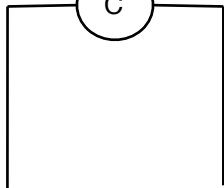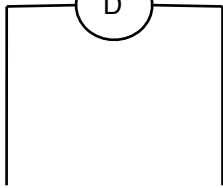

Supplement: Figure S5 — Calculation of over all tree height. Schematic representation of the summed branch-length tree height statistic. Overall tree height is computed by summing up the distance between all parents and child nodes. Note that the branch lengths of terminal branches (“leafs”) are not considered. Overall tree height = . (PDF) [file pone.0043689.s005.pdf]
