# Supplementary material for: Specific Genomic Regions Are Differentially Affected by Copy Number Alterations across Distinct Cancer Types, in Aggregated Cytogenetic Data
Source: PLoS One. 2012 Aug 24;7(8):e43689. doi: 10.1371/journal.pone.0043689 (PMC3427184; doi:10.1371/journal.pone.0043689)
Supplement: Figure S2 — Dendrogram of a permuted frequency matrix. For this clustering, the frequencies among cancer types were permuted and then normalized. Hierarchial Ward clustering was then performed and the dendrogram tree shown was obtained. The tree height is severely affected by the permutation. In this randomized clustering, similar cancer types no longer clustered together. (PDF) [file pone.0043689.s002.pdf]

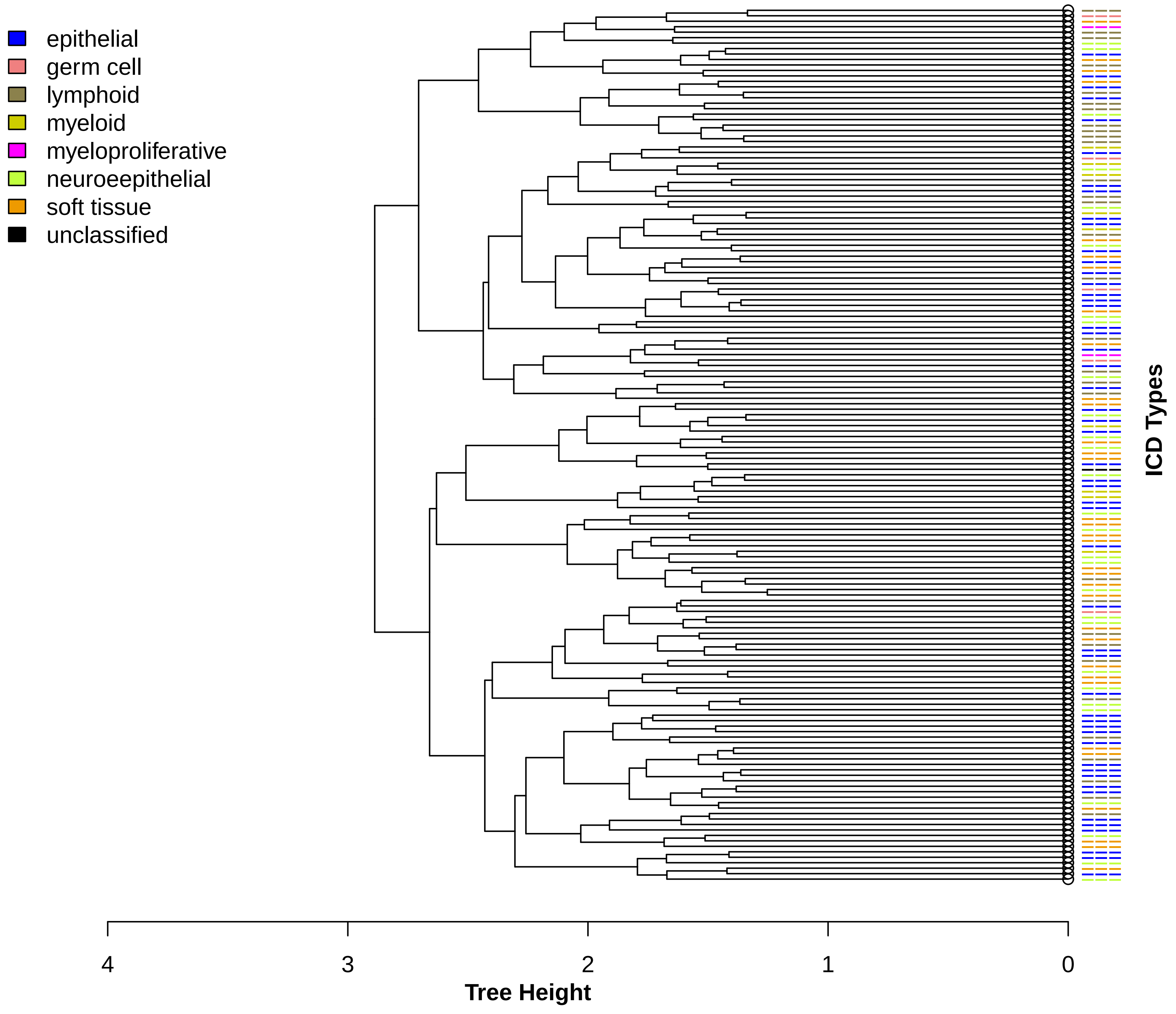
epithelial
germ cell
lymphoid
myeloid
myeloproliferative
neuroeepithelial
soft tissue
unclassified
ICD Types
4
3
2
1
0
Tree Height
