# Supplementary material for: Specific Genomic Regions Are Differentially Affected by Copy Number Alterations across Distinct Cancer Types, in Aggregated Cytogenetic Data
Source: PLoS One. 2012 Aug 24;7(8):e43689. doi: 10.1371/journal.pone.0043689 (PMC3427184; doi:10.1371/journal.pone.0043689)
Supplement: Figure S3 — Small regions from heatmap in main Figure 3 are shown here. These regions represent gains and losses on 7q and 8q. 8q changes differentiate between two categories of brain tumors, with a subset showing preferential losses on 8q (green labels) and other rarely showing involvement of 8q locus (red label). Thus depending on 8q involvement neuroepithelial tumors can be divided in to two different categories. Both of them show 7q gains. (PDF) [file pone.0043689.s003.pdf]

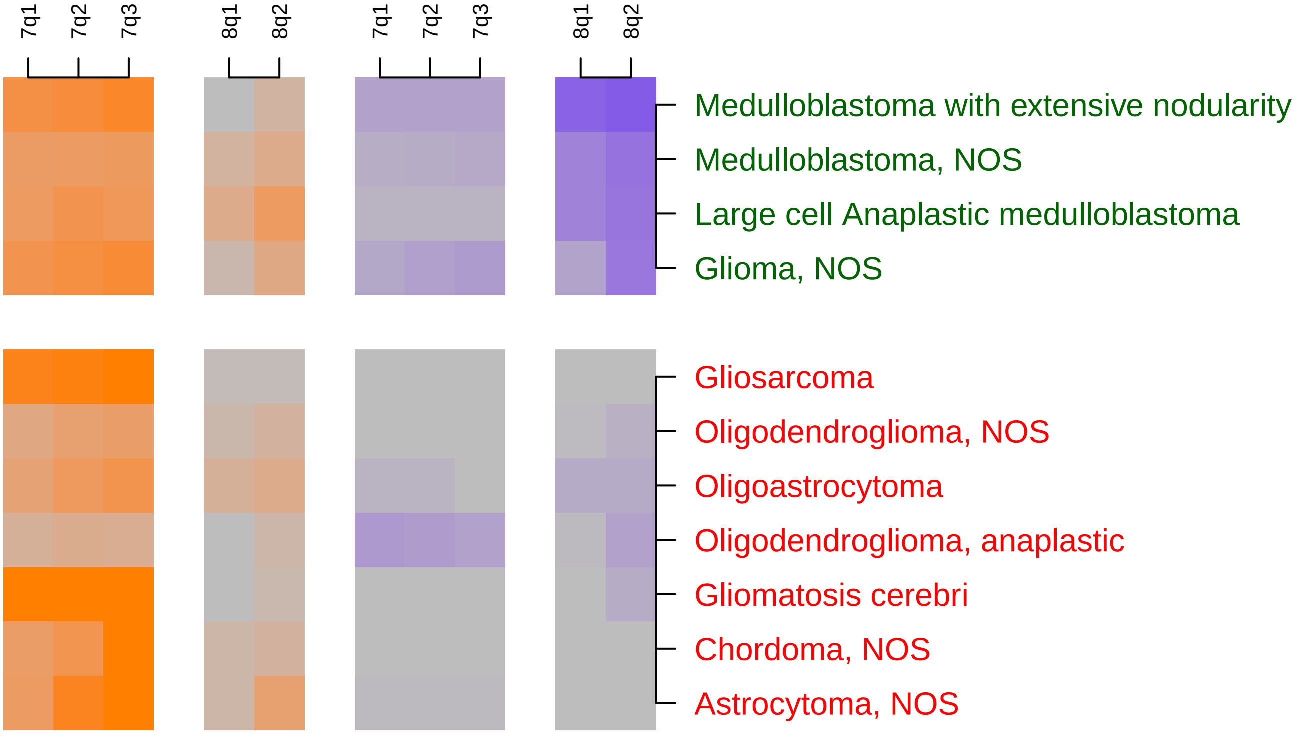

7q1
7q2
7q3
8q1
8q2
7q1
7q2
7q3
8q1
8q2
Medulloblastoma with extensive nodularity
Medulloblastoma, NOS
Large cell Anaplastic medulloblastoma
Glioma, NOS
Gliosarcoma
Oligodendroglioma, NOS
Oligoastrocytoma
Oligodendroglioma, anaplastic
Gliomatosis cerebri
Chordoma, NOS
Astrocytoma, NOS
