# Supplementary material for: Specific Genomic Regions Are Differentially Affected by Copy Number Alterations across Distinct Cancer Types, in Aggregated Cytogenetic Data
Source: PLoS One. 2012 Aug 24;7(8):e43689. doi: 10.1371/journal.pone.0043689 (PMC3427184; doi:10.1371/journal.pone.0043689)
Supplement: Figure S4 — Examples for non-neutral CNA regions. a) Heatmap of CNA profiles on genomic regions (same as in Figure 3). b) Small regions (red rectangles on the heatmap) are zoomed in to show how 8q is preferentially lost in in germ cell (black labels) tumors and is preferentially gained in epithelial cancer types (pink labels). c) Small regions (black rectangles on the heatmap) are zoomed in to show how 18q is preferentially gained in medullublastomas (brown labels) and is preferentially lost in epithelial tumors (pink labels). The examples here show that how two different non-neutral changes differential epithelial tumors from germ cell tumors and follicular lymphomas. (PDF) [file pone.0043689.s004.pdf]

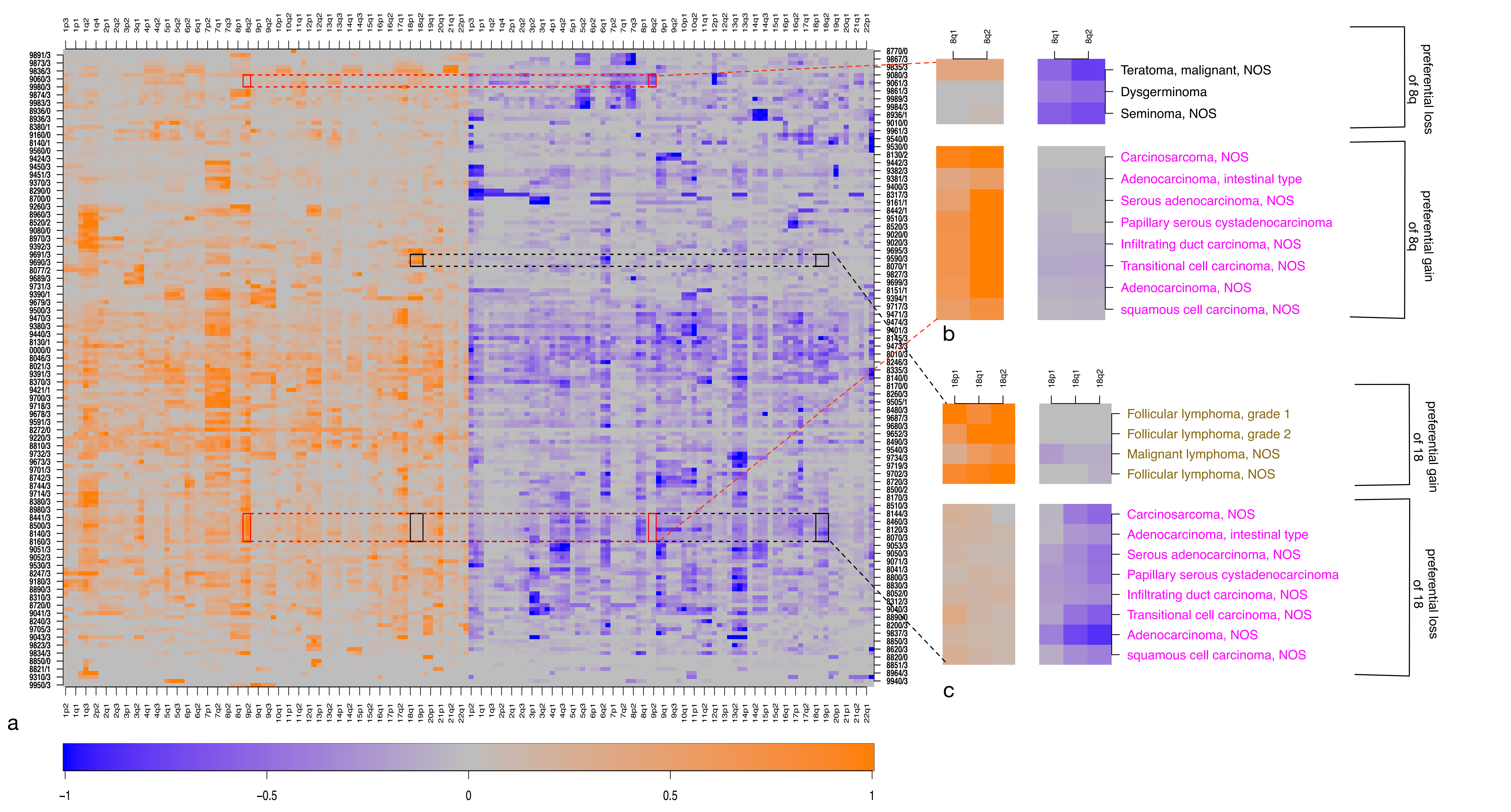

a
b
c
8q1
8q2
Teratoma, malignant, NOS
Dysgerminoma
Seminoma, NOS
preferential loss of 8q
Carcinosarcoma, NOS
Adenocarcinoma, intestinal type
Serous adenocarcinoma, NOS
Papillary serous cystadenocarcinoma
Infiltrating duct carcinoma, NOS
Transitional cell carcinoma, NOS
Adenocarcinoma, NOS
squamous cell carcinoma, NOS
preferential gain of 8q
18p1
18q1
18q2
Follicular lymphoma, grade 1
Follicular lymphoma, grade 2
Malignant lymphoma, NOS
Follicular lymphoma, NOS
preferential gain of 18
Carcinosarcoma, NOS
Adenocarcinoma, intestinal type
Serous adenocarcinoma, NOS
Papillary serous cystadenocarcinoma
Infiltrating duct carcinoma, NOS
Transitional cell carcinoma, NOS
Adenocarcinoma, NOS
squamous cell carcinoma, NOS
preferential loss of 18
−1
−0.5
0
0.5
1
